# Combined Apocynin and Novokinin Treatment Attenuates Renal Damage and Reduces KIM-1 Expression in a Model of Ischemic AKI

**DOI:** 10.3390/ijms27146345

**Published:** 2026-07-17

**Authors:** Milan Ivanov, Nevena Mihailović-Stanojević, Una-Jovana Vujačić, Danijela Karanović, Djurdjica Jovović, Maja Životić, Sanjin Kovacevic, Jelena Nešović-Ostojić, Zoran Miloradović

**Affiliations:** 1 Institute for Medical Research, National Institute of Republic of Serbia, University of Belgrade, Dr Subotića 4, 11000 Belgrade, Serbia; nevena@imi.bg.ac.rs (N.M.-S.); unajovana@imi.bg.ac.rs (U.-J.V.); danijela.karanovic@imi.bg.ac.rs (D.K.); djurdjica@imi.bg.ac.rs (D.J.); zokim@imi.bg.ac.rs (Z.M.); 2 Institute of Pathology, Faculty of Medicine, University of Belgrade, Dr Subotića 1, 11000 Belgrade, Serbia; majajoker@gmail.com; 3 Institute of Pathological Physiology, Faculty of Medicine, University of Belgrade, Dr Subotića 1, 11000 Belgrade, Serbia; sanjin.kovacevic@med.bg.ac.rs (S.K.); jelena.nesovic-ostojic@med.bg.ac.rs (J.N.-O.)

**Keywords:** acute kidney injury, apocynin, novokinin, NADPH oxidase, angiotensin II type 2 receptor, kidney injury molecule-1, hypertension

## Abstract

Ischemia–reperfusion injury (IRI) of the kidney represents a serious medical issue with complex pathological mechanisms that remain poorly elucidated. The development of ischemic acute kidney injury (AKI) may be mediated by NADPH oxidase and angiotensin II type 2 receptors. The aim of our study was to explore the effects of novokinin (NOV), angiotensin II type 2 receptor (AT2R) agonist and apocynin (APO), an NADPH oxidase inhibitor, on kidney structure and function, as well as the level of oxidative stress in spontaneously hypertensive rats (SHR) with induced IRI. Animals were randomly assigned into five experimental groups: the Sham-operated animals (SHAM) group, the AKI group, and AKI groups receiving APO, NOV, or their combination. Slight improvements in renal function were noted across all treated groups; however, only the combined action of APO + NOV led to a significant decrease in kidney injury molecule-1 (KIM-1) levels and markedly reduced oxidative stress compared with the control AKI group. Our results demonstrate that additively targeting NADPH oxidase and AT2R provides new insights in comparison to individual treatments. These findings suggest a potentially promising new therapeutic strategy for managing IRI and improving outcomes in critically ill patients with AKI.

## 1. Introduction

Acute kidney injury (AKI) is a common clinical event. It affects around 20% of emergency-admitted patients. Contemporary series report mortality rates of around 25–40%. In humans, pre-existing conditions like hypertension or chronic kidney disease heavily exacerbate the severity of AKI, and when associated with hypertension, it causes even higher mortality rates [1,2]. The spontaneously hypertensive rat (SHR) model develops these comorbidities naturally, making it a highly relevant translational model for studying AKI in vulnerable populations. Renal ischemia–reperfusion (I/R) injury (IRI) is one of the most common causes of AKI. It occurs for several reasons, such as blood loss after surgery and trauma, the use of vasoconstrictive drugs or radiocontrast agents, and sepsis [2]. Renal IRI leads to a rapid decline in renal function due to a restricted blood supply, followed by the restoration of blood flow and reoxygenation. IRI aggravates tissue damage by initiating an inflammatory cascade, including the production of reactive oxygen species (ROS), cytokines, and chemokines, as well as leukocyte activation [3]. Unfortunately, therapeutic approaches to prevent or treat renal IRI are extremely limited; thus, all efforts are made to detect AKI early and establish secondary preventive measures to inhibit its progression [4].

Clinical and experimental studies reveal prominent activation of the renin–angiotensin–aldosterone system in AKI, leading to increased angiotensin II (Ang II) formation in the kidney tissue [5,6]. Ang II, as one of the main vasoactive signaling molecules, is involved in ROS generation. Overproduction of Ang II during renal IRI [7,8] participates in the increased expression and activity of one of the major ROS generators, NADPH oxidase (Nox) [9].

The overwhelmed capacity of the antioxidant defense system during reperfusion injury suggests that the main target of IRI therapies should be a pharmacological approach to decrease renal oxidative stress. A previous study [6] showed that the blockade of angiotensin II type 1 receptors (AT1R) decreases oxidative stress and improves renal hemodynamics. One possible reason for this reaction is the binding of free Ang II to angiotensin II type 2 receptors (AT2R) [10] and the simultaneous inhibition of Nox enzyme activation pathways [11].

Apocynin (APO, 4-hydroxy-3-methoxyacetophenone) is an efficient inhibitor of Nox. The antioxidant and anti-inflammatory effects of Nox inhibition involve the impairment of the intracellular translocation of two critical cytosolic components of the Nox complex present in the cell membrane. They also involve activation by myeloperoxidase (MPO) because agents that promote the release of this enzyme enhance the efficacy of APO, whereas Nox inhibition is absent in cells lacking or deficient in myeloperoxidase [12].

Novokinin (NOV) is an angiotensin AT2R agonist (Ki = 7.35 μM) that displays 93-fold selectivity over AT1. It exhibits vasorelaxing and hypotensive effects via activation of the IP (prostacyclin) receptor [13]. Although AT2R was discovered over two decades ago, its role in physiology and pathophysiology remains incompletely elucidated. Padia and Carey [10] explain that AT2R represents a beneficial counter-regulatory mechanism that protects the kidney from ischemic injury, and that AT2R activation also protects the kidney from inflammatory changes induced by AT1R overexpression. AT2R activation can be established as an important part of therapeutic strategies to prevent and/or reverse tissue damage due to reperfusion injury occurring during ischemic AKI.

Numerous studies have demonstrated that losartan, through AT1R blockade, attenuates Ang II-induced oxidative stress, partly by reducing Nox activity, while potentially shifting Ang II signaling toward the AT2R pathway [9,10]. Namely, due to AT1R blockade, losartan inhibits the Nox activation pathway [9]. On the other hand, a high concentration of free Ang II (produced during renal ischemia) stimulates the protective AT2R cascade [10]. In this context, we hypothesize that the same effects could be obtained when a Nox inhibitor is combined with a selective AT2R agonist in downstream pathways.

Considering the above, the aim of this study was to evaluate the early renoprotective effects of single or combined treatment with the AT2R agonist novokinin and the Nox inhibitor apocynin, administered immediately before reperfusion, on kidney structure, function, and oxidative stress in SHRs subjected to renal I/R injury.

## 2. Results

### 2.1. Haemodynamic Parameters

Systemic hemodynamic parameters, 24 h after reperfusion, are shown in Table 1. The MAP was significantly decreased in the AKI group compared with the SHAM group. Furthermore, in all treated groups, MAP was significantly increased in comparison to the AKI group, but only in the NOV group was it non-significantly different from SHAM. HR was significantly lower in all AKI groups in comparison with SHAM. CO was significantly higher in both NOV-treated groups, but only the group with combined treatment showed a lower TPVR in comparison with the SHAM and AKI groups.

**Figure 1:**
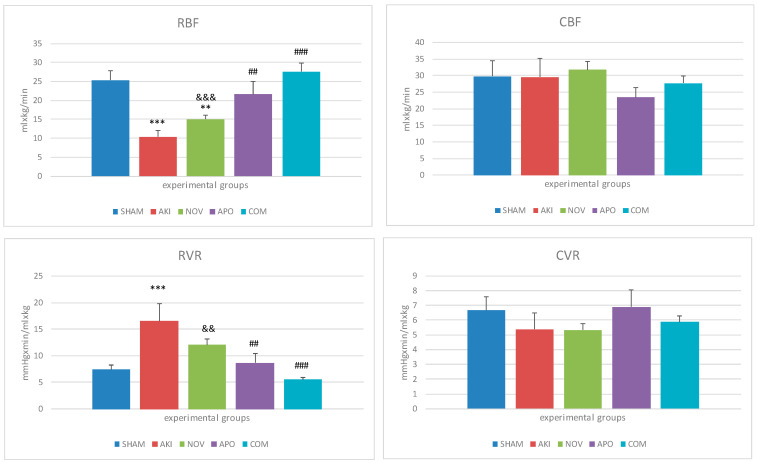
Renal and carotid haemodynamic parameters, 24 h after reperfusion. RBF—Renal blood flow; CBF—Carotid blood flow; RVR—Renal vascular resistance; CVR—Carotid vascular resistance; SHAM (*n* = 7)—Sham-operated rats (model control group); AKI (*n* = 7)—control group with acute kidney injury; NOV (*n* = 9)—animals with AKI and received novokinin; APO (*n* = 9)—animals with AKI and received apocynin; COM (*n* = 9)—animals with AKI and received NOV and APO; ** *p* < 0.01, *** *p* < 0.001, vs. SHAM group; ^##^ *p* < 0.01, ^###^ *p* < 0.001 vs. AKI group; ^&&^ *p* < 0.01, ^&&&^ *p* < 0.001 vs. COM group. The results are expressed as mean ± standard error of the mean.

The significant decline in RBF in the AKI group and the concomitant increase in RVR (compared with SHAM) were completely abolished with the combination of NOV and APO. Namely, this combination markedly increased RBF and decreased RVR in hypertensive rats with AKI (Figure 1). While individual treatments improved renal hemodynamics, their combination resulted in a much greater benefit on these parameters. There were no differences in CBF and CVR between groups.

### 2.2. Kidney Function Parameters

**Figure 2:**
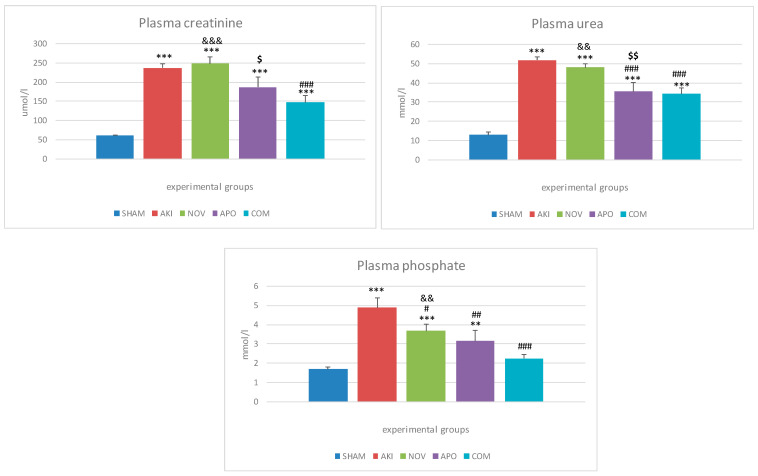
Kidney Function Parameters (Pcr, Pu, Pphos) in experimental groups. SHAM (*n* = 7)—Sham-operated rats (model control group); AKI (*n* = 7)—control group with acute kidney injury; NOV (*n* = 9)—animals with AKI and received novokinin; APO (*n* = 9)—animals with AKI and received apocynin; COM (*n* = 9)—animals with AKI and received NOV and APO; ** *p* < 0.01, *** *p* < 0.001, vs. SHAM group; ^#^ *p* < 0.05, ^##^ *p* < 0.01, ^###^ *p* < 0.001 vs. AKI group; ^&&^ *p* < 0.01, ^&&&^ *p* < 0.001 vs. COM group; ^$^ *p* < 0.05, ^$$^ *p* < 0.01 vs. NOV group. The results are expressed as mean ± standard error of the mean.

Plasma creatinine (Pcr), urea (Pu) and phosphate (Pphos) levels were measured to assess renal function (Figure 2). Significantly higher levels of Pcr, Pu and Pphos were found in the AKI group in comparison to the SHAM group. Kidney function was improved only in the COM group after combined treatment with APO and NOV, as evidenced by significant decreases in Pcr, Pu, and Pphos levels. Single treatments with APO or NOV slightly, but not significantly, improved Pcr, while only APO significantly improved Pu. On the other hand, both single treatments reduced Pphos levels.

### 2.3. Kindy Injury Molecule-1 (KIM-1) in Plasma

**Figure 3:**
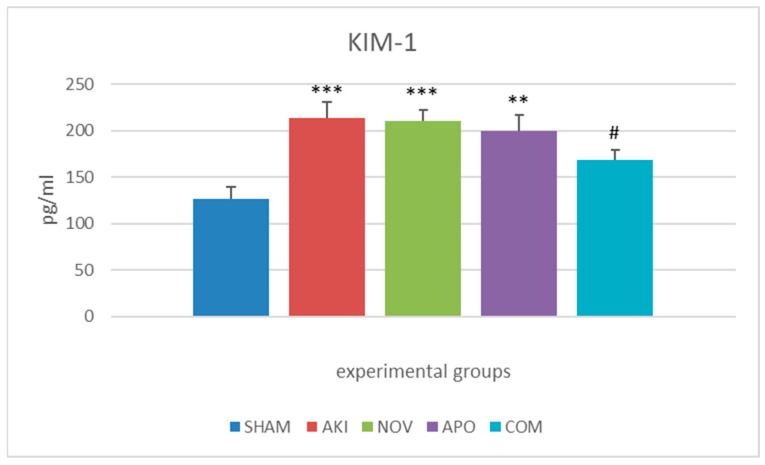
Plasma kidney injury molecule-1 (KIM-1) levels in experimental groups. SHAM (*n* = 7)—Sham-operated rats (model control group); AKI (*n* = 7)—control group with acute kidney injury; NOV (*n* = 9)—animals with AKI and received novokinin; APO *(n* = 9)—animals with AKI and received apocynin; COM (*n* = 9)—animals with AKI and received NOV and APO ** *p* < 0.01, *** *p* < 0.001, vs. SHAM group, ^#^ *p* < 0.05. vs. AKI group. The results are expressed as mean ± standard error of the mean.

KIM-1 levels in plasma, 24 h after reperfusion, are shown in Figure 3. After AKI induction, KIM-1 levels were significantly increased in the AKI group as compared to the SHAM group. Combined treatment with APO and NOV, in contrast to single treatment with either of these substances, significantly decreased the level of KIM-1 in plasma compared to the AKI group.

Furthermore, KIM-1 levels were significantly positively correlated with renal function parameters (Table 2).

### 2.4. Oxidative Stress Parameters

Catalase (CAT) and lipid peroxidation levels in plasma are important indicators of antioxidant defense capacity and oxidative injury, and they are useful for assessing kidney damage. Rats with AKI exhibited a significant increase in lipid peroxidation compared with sham-operated animals. COM treatment reduced I/R AKI-induced plasma overproduction of TBARS to a level not significantly different from SHAM (*p* = 0.204). The level of TBARS in groups treated with NOV or APO alone showed no improvement compared to AKI.

Superoxide dismutase (SOD), glutathione peroxidase (GSH-Px), and glutathione reductase (GR) activities were not different between groups (Table 3). The AKI group showed a significant decrease in CAT activity compared to sham-operated animals. Only combined therapy resulted in a significant increase in CAT, in contrast to single treatment with APO or NOV, compared to AKI alone (Table 3).

### 2.5. Histological Studies

**Figure 4:**
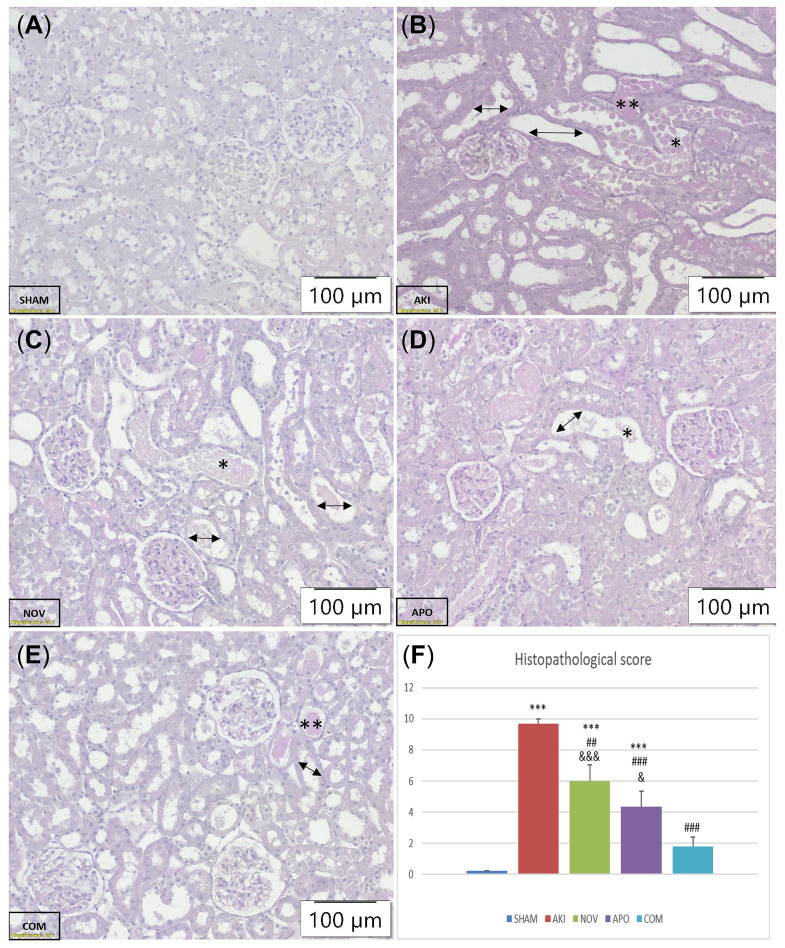
Histopathology of the representative kidney samples collected in different experimental groups (PAS staining, ×20 magnification): Normal morphology of renal tissue including glomerular and tubulointerstitial compartments in the sham-operated animals (**A**), proximal tubular dilatation (arrows), necrosis of tubular epithelial cells (*) and PAS-positive casts (**) in the AKI group (**B**), moderately intensive tubular necrosis, reduced tubular dilatation and less number of PAS-positive casts in NOV (**C**), APO (**D**) and COM (**E**) groups, and histopathological score (**F**) as a sum of present morphological changes. *** *p* < 0.001 vs. SHAM group; ^##^ *p* < 0.01, ^###^ *p* < 0.001 vs. AKI group; SHAM (*n* = 7)—Sham-operated rats (model control group); AKI (*n* = 7)—control group with acute kidney injury; NOV (*n* = 9)—animals with AKI and received novokinin; APO (*n* = 9)—animals with AKI and received apocynin; COM (*n* = 9)—animals with AKI and received NOV and APO; ^&^ *p* < 0.05, ^&&&^ *p* < 0.001 vs. COM. The results are expressed as mean ± standard error of the mean.

The light microscopic findings of the kidney are shown in Figure 4. Significant differences in pathomorphological parameters existed between the experimental groups. Figure 4A shows the normal appearance of glomeruli, interstitium, tubules, and blood vessels in SHAM-operated animals. In only a few kidney specimens, we observed a small number of PAS-positive casts in the lumen of the tubules. The kidneys of animals with AKI showed dilatation of certain segments of the proximal and distal tubules, with or without loss of the brush border. The most notable changes were present in the corticomedullary zone, where broad areas of tubular necrosis and a large number of PAS-positive casts in the collecting ducts were observed. The intensity of interstitial edema in this group varied from sample to sample (Figure 4B). In treated animals (APO, NOV, and COM), significantly fewer morphological changes were noticed compared to the AKI control, with reduced tubular dilatation, tubular necrosis in the corticomedullary zone, and PAS-positive cast formation (Figure 4C–E). In addition, the most significant improvement in kidney morphology was observed in the group with combined treatment. The histopathological score (Figure 4F), as a sum of these changes, was significantly higher in the AKI group compared to the SHAM control. In the treated groups, this score was significantly lower in comparison to AKI.

## 3. Discussion

Our previous research [6,14] showed that losartan treatment, due to AT1R blockade, indirectly affects AT2R [10] and Nox [15]. For this reason, in the present study, we investigated the effects of AT2R activation and Nox inhibition on I/R-induced AKI development in hypertensive rats. We demonstrate, for the first time, the importance of additive AT2R stimulation and Nox blockade in blunting the development of AKI.

We evaluated hemodynamic parameters, kidney function, oxidative status, and kidney morphological changes 24 h after reperfusion.

Hemodynamic parameters observed in the present study are in accordance with our previous findings [6], confirming that MAP is reduced after AKI induction. In patients, reductions in systolic blood pressure decrease organ perfusion, including renal perfusion, and contribute to AKI [16]. Considering data from the literature, decreased blood pressure could be a consequence of azotemia/uremia levels following the development of postischemic kidney injury [17].

Treatment with NOV abolished the AKI-induced hypotensive effect. This result confirms the hypothesis of Danyel et al. about the role of AT2R in blood pressure regulation [18]. Namely, they suggest that AT2R agonists are not suitable as antihypertensives, but they can improve protection from hypertension-induced end-organ damage. Böhm et al. showed that HR can play an important role in predicting the development of renal disease. Their study suggests that lowering HR could have a protective role in kidney preservation [19]. In our study, all groups with induced AKI showed HR responses toward kidney preservation.

There are numerous studies that point to a correlation between AKI and the development of cardiorenal syndrome [19,20]. These authors emphasize the importance of cardiac output. This parameter is especially important if AKI is accompanied by comorbidities, such as hypertension. Viswanathan and Gilbert reported in their study that agents such as inotropes may be effective in restoring kidney function by augmenting cardiac output [20]. In our study, we showed, for the first time, that AT2R stimulation increases CO and reduces TPVR in SHRs with I/R AKI. Additionally, simultaneous AT2R stimulation and NADPH oxidase blockade result in a greater increase in CO, accompanied by a corresponding decrease in peripheral resistance. Furthermore, it is important to acknowledge that the observed renoprotection may, in part, be secondary to these systemic circulatory changes. This improvement in systemic hemodynamics (indicated by MAP, CO, and TPVR) could directly contribute to the normalization of RBF and RVR, thereby enhancing overall renal perfusion. Namely, twenty-four hours after I/R AKI induction, RBF was significantly reduced, followed by enhanced RVR in SHRs. Intrarenal vasoconstriction is one of the main factors involved in the initiation and maintenance of AKI [14,21], and a selective AT1R antagonist, losartan, significantly increased RBF and reduced RVR in I/R AKI. Miloradović et al. [21] showed that losartan (10 mg/kg b.w.) strongly increased RBF following moderate renal NO deficiency in postischemic Wistar rats. The results of our study are consistent with theirs. Namely, in the study of Miloradović et al., blockade of AT1R in postischemic AKI, on the one hand, allows free Ang II to bind to AT2R, and on the other hand, prevents the activation of NADPH oxidase [15]. Our results indicate that Nox inhibition plays a major role, but only the additive effect of Nox blockade and AT2R stimulation can achieve a complete beneficial effect on renal hemodynamics during AKI development.

AKI is characterized by a rapid deterioration in renal function. Pcr and Pu were used as measures of the glomerular filtration rate (GFR). GFR is estimated via plasma creatinine, with the understanding that a twofold elevation in baseline creatinine (e.g., 50 to 100 μmol/L) corresponds directly to a halving of the GFR [22]. In our study, I/R induced a fourfold rise in Pcr and Pu in the model AKI group, indicating rapid GFR deterioration in hypertensive kidneys subjected to AKI. Only the additive action of APO and NOV significantly lowers levels of creatinine and urea in plasma 24 h after reperfusion in SHRs. Ciarcia et al. [23] showed that APO may have a beneficial effect on GFR in a model of cyclosporine A-induced hypertension and nephrotoxicity in rats. Numerous studies have highlighted the importance of AT2R in renal hemodynamic regulation and the preservation of kidney function [24,25]. Hilliard et al. [25] showed that AT2R stimulation can be a novel target for ameliorating renal disease, but without affecting GFR (similar to our study). However, for the first time, we showed that combining NADPH oxidase blockade with AT2R stimulation significantly improved kidney function and increased GFR in an experimental model of AKI and hypertension.

The kidney plays a major role in phosphate homeostasis, which can be disrupted in patients with kidney disease. Hyperphosphataemia usually develops in postischemic AKI [14,26]. Besides that, hyperphosphatemia is associated with increased risks of end-stage renal disease and mortality, and it may therefore be necessary to monitor serum phosphorus levels in hospitalized patients, irrespective of kidney function [27]. According to Rubinger et al. [28], downregulation of the renal sodium-dependent phosphate cotransporter NaPi_2_ following I/R could account for decreased tubular phosphate reabsorption and hyperphosphatemia. In our study, AKI-induced hyperphosphataemia was successfully repressed by single treatments with APO or NOV, as well as by their combination. However, it should be emphasized that the best result was obtained with the combined treatment. Combined treatment decreases Pphos levels to almost the level of the SHAM-operated group.

Homeostasis is favored by balancing the production of both oxidants and antioxidants. It has been proven that ROS play a physiological role in renal function control, but when reactive species are unregulated or upregulated, leading to local accumulation, they can induce oxidative stress, irreversibly damaging DNA, RNA, lipids, and proteins, and causing organelle dysfunction [29]. A common link between all forms of AKI, regardless of cause, is the enhanced generation of ROS during injury/disease progression. IRI represents a major cause of AKI, and the main cause of delayed renal graft function and renal graft loss after kidney transplantation [30]. In IRI, the reperfusion phase is the critical moment when the most IRI damage might occur. The initial event that occurs immediately after reperfusion is a sudden increase in mitochondrial superoxide anion production, which is released into the cell and serves as the main trigger for the injury that follows reperfusion [31,32,33]. Therefore, the main target of IRI therapies should be novel approaches to reduce renal oxidative stress. The roles of Ang II and NADPH oxidase in the development of oxidative stress in pathophysiological conditions, such as AKI, are unequivocal [11,34,35]. Therefore, the focus of this study is the roles of AT2R and NADPH oxidase in the oxidative status of SHRs during the development of AKI.

The ROS production is usually counterbalanced by the formation of endogenous antioxidants, which disrupt the damaging effects of oxidative stress. However, in the case of AKI, endogenous antioxidants are insufficient to counteract increased ROS production [6,36,37]. In this situation, exogenous antioxidants or therapeutics that increase antioxidant defence would be useful in treating this disease.

Many studies show elevated lipid peroxidation after renal ischemia [6,36,38]. In our study, only the combined treatment has a beneficial effect on lipid peroxidation, with a decrease after AKI development. Namely, only the combined action of APO and NOV did not lead to an increase in lipid peroxidation. It is well known that AT2Rs are upregulated in cardiovascular disease at the site of tissue injury [39] and for that reason, the potential of AT2R as a possible therapeutic target cannot be underestimated. In accordance with our findings, the effects of AT2R are generally more subtle and often are unmasked when the dominant AT1R effects are inhibited. Namely, our previous study [6] showed beneficial effects of AT1R blockade on lipid peroxidation in an experimental model of I/R AKI induced in SHRs. This benefit could have been the result of concomitant NADPH oxidase blockade from AT1R antagonism and AT2R stimulation from increased plasma Ang II levels. In accordance with this, our study showed that individual actions of NADPH oxidase blockade or AT2R receptor stimulation do not confer a beneficial effect on lipid peroxidation, in contrast to their additive effects.

A lower level of TBARS is directly correlated with increased catalase activity [40]. Actually, increased CAT activity leads to a decrease in TBARS levels in the experimental groups. Similar results were obtained in our previous studies, where we showed that (in the same model) a decrease in lipid peroxidation in plasma is always accompanied by increased CAT activity, as well as significantly better kidney function and structure [6,38,41].

Ritz and Haxen [15] showed that the AngII-AT1R-mediated effects on kidney ROS were associated with NADPH oxidase overexpression in SHRs. Koh et al. [42] showed that the AT1R antagonist, candesartan, decreased oxidative stress and significantly attenuated lipid peroxidation in humans. Our previous study [6] showed that, despite high levels of Ang II due to ischemic kidney damage, AT1R blockade reduced oxidative stress and improved morphological changes in the damaged kidney. In that model, we assume that during AT1R blockade, free Ang II stimulates AT2R, thereby improving ischemic kidney function.

The decreased level of GSH-Px activity in SHRs with I/R AKI after combined therapy can be explained by the significantly increased level of CAT. Namely, CAT and GSH-Px are equally involved in the removal of H_2_O_2_ in human erythrocytes [43]. Furthermore, in astrocytes, GSH-Px and catalase could functionally substitute for each other in H_2_O_2_ detoxification [44].

The lack of significant alterations in erythrocyte SOD, GSH-Px, and GR activities likely stems from our 24-h post-reperfusion timeline, where systemic erythrocyte pools may not fully capture the acute, localized intrarenal oxidative dynamics. Additionally, the prominent upregulation of CAT in the COM group may have provided sufficient peroxide detoxification, reducing the immediate compensatory demand on alternative enzymatic pathways like GSH-Px [45].

Kidney Injury Molecule-1 (KIM-1) is a type 1 transmembrane protein, with an immunoglobulin and mucin domain, whose expression is markedly up-regulated in the proximal tubule after post-ischemic kidney injury. Therefore, KIM-1 might serve as a blood or urine biomarker of acute renal tubular injury [46]. Sabbisetti et al. [47] demonstrated that plasma KIM-1 levels progressively increase in proportion to the extent of renal ischemia–reperfusion injury. This trend was also confirmed in our study. Moreover, plasma KIM-1 showed a strong positive correlation with conventional markers of renal dysfunction, including serum creatinine, urea, and phosphate levels. These findings support the role of plasma KIM-1 as a reliable biomarker of worsening kidney function in AKI [48].

Sen et al. showed that in patients with type 2 diabetes, lowering plasma KIM-1 levels reduces the risk of kidney disease progression [49]. Consistent with these findings, our study also demonstrates that a significant decrease in plasma KIM-1 in the APO + NOV group is associated with improved renal function. Furthermore, all these associations were verified by pathohistological examinations.

The lack of significant efficacy in the single-treatment groups (NOV or APO alone) regarding creatinine, TBARS, and KIM-1 highlights the limitations of monotherapy in ischemic AKI. Blocking NADPH oxidase alone leaves alternative Ang II-mediated damage unchecked, while stimulating AT2R is overwhelmed by ongoing ROS production if NDPH oxidase remains active. These negative outcomes demonstrate that individual interventions cannot disrupt this tightly integrated loop, confirming the absolute necessity of a simultaneous, dual-target approach to achieve renoprotection.

Numerous studies indicate potential renoprotective effects of NADPH oxidase blockade or AT2 receptor stimulation [37,49,50,51,52,53,54,55]. Tan et al. [50] showed that oxidase blockade can be an important part of the strategy to preserve renal function in nephrotoxic kidney injury. However, although APO is commonly used as a Nox inhibitor, its effects should be interpreted with caution, since it is not a direct and fully selective inhibitor of vascular Nox. Its inhibitory action depends largely on MPO-mediated activation and is therefore more pronounced in MPO-expressing leukocytes, whereas vascular cells with low MPO activity may not adequately activate APO. However, in the context of I/R-induced AKI, this limitation may be less critical during the reperfusion phase, which is characterized by neutrophil recruitment and neutrophil-derived ROS production. Therefore, APO may still effectively attenuate oxidative stress during reperfusion, primarily by targeting Nox activity in infiltrating MPO-expressing neutrophils, rather than by directly inhibiting vascular Nox. Moreover, part of the beneficial effect of APO may be mediated indirectly, through the attenuation of oxidative stress-driven inflammation and improvement of renal microvascular function, rather than through selective inhibition of vascular Nox alone [37,41]. On the other hand, Matavelli et al. [51] claim that AT2 receptor stimulation reduces inflammation and improves the structure of the ischemic kidney in hypertensive two kidney one clip (2K1C) rats. These claims are supported by our results, indicating that the combined treatment with APO and NOV leads to a significant improvement in renal structure and function. In our study, individual treatments show a positive trend in improving kidney function, but only when applied simultaneously, APO and NOV results in visible kidney recovery as assessed by histological findings.

To ensure a balanced interpretation of our findings, we acknowledge the following limitations, which also provide directions for future research:

- Although the combined treatment showed clear functional, biochemical, and histological benefits, additional mechanistic studies are required to further clarify the precise molecular interactions between NADPH oxidase inhibition and AT2 receptor stimulation in ischemic AKI.
- This investigation utilized male SHRs exclusively. Because AT2R expression, density, and downstream signaling cascades are significantly modulated by sex hormones, one limitation of the current study is that the results cannot be broadly extrapolated to females without dedicated future trials.
- The unilateral nephrectomy combined with contralateral renal artery occlusion model is a standard approach used in preclinical research to study AKI. It is highly reproducible in laboratory settings but introduces several limitations when translating experimental findings to human patients. The loss of natural repair “crosstalk’’ (which exists when one kidney is totally damaged and the other is intact) resulted in losing influence on immune, inflammatory, and cellular repair pathways in the injured organ.
- Our study endpoint was restricted to 24 h post-reperfusion. While this period successfully captures the peak of the acute injury phase and initial cellular necrosis, it provides no data regarding long-term recovery trajectories, chronic fibrotic progression, or the sustainability of the combination therapy’s protective effects.
- A further limitation is that both treatments were administered immediately before reperfusion and therefore evaluated early peri-reperfusion protection rather than the therapy of established AKI. Consequently, these findings may not be directly transferable to clinical settings in which treatment begins only after renal dysfunction becomes evident. Nevertheless, this design was intended to model high-risk procedures, including cardiothoracic, major vascular, and transplantation surgery, in which renal ischemia and reperfusion can be anticipated and preventive intervention immediately before reperfusion may be clinically feasible.

## 4. Materials and Methods

In our study, we used 24-week-old male SHRs, weighing about 300 g, bred at the Institute for Medical Research, University of Belgrade, Serbia. The rats were housed under standard conditions (temperature 23 ± 2 °C, humidity 55 ± 10%, and 12 h light/dark cycle) in plastic cages (four rats per cage) with free access to water and standard chow for laboratory rats (AGRO-FIRM doo, Požarevac, Serbia).

### 4.1. Ethics Statement

The experimental protocol was approved by the Ethical Committee of the Institute for Medical Research, University of Belgrade, as well as the Veterinary Directorate, Ministry of Agriculture and Environmental Protection, Serbia (No. 323-07-02569/2018-05/1), in accordance with the National Law on Animal Welfare (“Službeni Glasnik” no. 41/09, 39/10), that is consistent with guidelines for animal research and principles of the European Convention for the Protection of Vertebrate Animals Used for Experimental and Other Purposes (Official Daily N. L 358/1-358/6, 18 December 1986) and Directive on the protection of animals used for scientific purposes (Directive 2010/63/EU of the European Parliament and of the Council, 22 September 2010).

### 4.2. Experimental Protocol

AKI was induced in an anesthetized (35 mg/kg b.w. sodium pentobarbital) adult male SHR by right kidney nephrectomy and a traumatic occlusion of the left renal artery for 40 min. After the clamp was removed, kidney reperfusion was confirmed visually.

Animals were divided into 5 experimental groups: Sham-operated rats (SHAM, *n* = 7), AKI control group (AKI, *n* = 7), rats given AT2R agonist NOV (Sigma Aldrich, St. Louis, MO, USA) after AKI induction (NOV, *n* = 9), rats given NADPH oxidase inhibitor APO (Sigma Aldrich) after AKI induction (APO, *n* = 9) and a combination (COM) group (*n* = 9) that received both substances after AKI induction.

SHAM and AKI rats received a vehicle (saline, 0.5 mL) bolus, while the NOV group received NOV (0.3 mg/kg b.w.) [13], APO group received APO (40 mg/kg b.w.) [41] and COM received both substances (in the same dose as a single treatment groups) dissolved in 0.5 mL saline, 5 min before renal artery clamp removal. Following the establishment of renal reperfusion, the abdominal incision was closed with several sutures and SHR were placed into metabolic cages for a 24 h recovery period, with free access to water and chow. To alleviate postoperative pain, ketoprofen (5 mg/kg b.w.) was administered subcutaneously.

### 4.3. Haemodynamic Measurements 24 h After Reperfusion

After 24 h, all animals were anesthetized (35 mg/kg b.w. sodium pentobarbital, i.p.). Haemodynamic parameters were measured through a femoral artery catheter (PE-50, Clay-Adams, Parsippany, NY, USA) connected to a physiological data acquisition system (9800TCR Cardiomax III-TCR, Columbus, OH, USA). A jugular vein was cannulated with polyethylene tubing PE-50 for the injection of cold saline. The left carotid artery was catheterized with PE-50 tubing and attached to a thermo sensor, which was coupled to the Cardiomax III for the determination of cardiac output (CO). The other end of the thermocouple was placed in cold saline. Following 20 min for stabilization after surgery, cold saline (0.2 mL) was supplied through the jugular vein and mean arterial pressure (MAP), heart rate (HR) and CO were recorded. Total peripheral vascular resistance (TPVR) was calculated from MAP and CO (assuming that mean right atrial pressure is zero) and expressed as mmHg min kg/mL.

For blood flow measurement, the left carotid artery was gently separated. An ultrasonic flow probe (1RB, internal diameter = 1 mm) was placed around the artery for the measurement of carotid blood flow (CBF) using a Transonic T106 Small Animal Flowmeter (Transonic System Inc., Ithaca, NY, USA). After abdominal incision, left renal artery preparation was utilized, and renal blood flow (RBF) was recorded. Vascular resistance in these two vascular beds (CVR and RVR) was calculated by dividing MAP by total blood flow through their respective blood vessels, normalized for body weight and expressed as mmHg × min × kg/mL.

### 4.4. Biochemical Measurements 24 h After Reperfusion

After haemodynamic measurements, blood samples were taken for the determination of Pcr, Pu and Pphos concentrations in plasma. Lithium-heparin (Sigma-Aldrich, St. Louis, MO, USA) was used as an anticoagulant. Samples were centrifuged at 4000 rpm, 4 °C, for 20 min, and plasma was stored at −20 °C until further analysis. All biochemical parameters were calculated using an automatic COBAS INTEGRA 400 plus (Hoffmann-La Roche, Leitch Diagnostic, Penzberg, Germany) analyzer. At the end of the experiment, all rats were sacrificed by a pentobarbital overdose injection according to the guidelines.

Plasma KIM-1 was determined by a sandwich enzyme-linked immunosorbent assay (Rat KIM-1 ELISA Kit, Cat. No. E-EL-R3019, Elabscience Biotechnology Co., Ltd., Houston, TX, USA). Plasma samples were prepared according to the manufacturer’s instructions. The detection range for KIM-1 was 0.31~20 ng/mL with a sensitivity of 0.19 ng/mL.

### 4.5. Oxidative Stress Parameters

To determine the degree of lipid peroxidation (LP), the concentration of thiobarbituric acid reactive substances (TBARS) in plasma was measured [41]. Erythrocyte catalase (CAT), glutathione reductase (GR), superoxide dismutase (SOD), and glutathione peroxidase (GSH-Px) activities were measured by spectrophotometry (Biochrom Ltd., Ultrospec 3300 pro, Cambridge, UK), according to previously described methods [6].

### 4.6. Morphological Examination

Immediately after removal, the left kidney tissue was fixed in 4% formaldehyde, rinsed in a series of ascending alcohol concentrations to dehydrate the tissue, and embedded in paraffin. Sections with a thickness of 5 µm were cut as described previously [6] and stained by periodic acid-Schiff (PAS). Intensity and spread of tubular necrosis, number of intra-luminal cast formations, swelling and vacuolization of cells, loss of luminal membrane or brush borders, tubular dilatation, interstitial oedema and separation of cells from tubular basal membrane were semi-quantitatively evaluated as described previously [6]. The level of each manifestation was graded as 1 for low, 2 for moderate, 3 for high, and 0 for a lack of manifestation. The sum of these changes represented the histopathological score. The slides were evaluated using an Olympus BX53 light microscope (Olympus Corporation, Tokyo, Japan).

### 4.7. Statistical Analysis

We performed a sample size calculation for a one-way analysis of variance (StatSoft, Inc. (2014). STATISTICA (data analysis software system), version 12. www.statsoft.com), which showed that the required total sample size for this study is 41 rats. Therefore, the rats were divided into 5 groups: 2 control groups (SHAM and model) with 7 animals per group and 3 treated groups with 9 animals per group (total *n* = 41). The results are expressed as mean ± standard error of the mean (SEM). The normality of data distribution was assessed using the Shapiro–Wilk test. One-way analysis of variance (ANOVA) was applied. When the ANOVA results were significant, the least significant difference (LSD) test was used as a post hoc multiple comparison test (Statistics for Windows). Correlation analysis was performed using Pearson’s correlation coefficient. *p*-values < 0.05 were considered significant.

## 5. Conclusions

Our findings demonstrate, for the first time, that combined treatment with APO and NOV appears to offer notable kidney protection against IRI-induced AKI, likely by combining NADPH oxidase inhibition with AT2 receptor stimulation. In contrast to individual treatments, this dual approach produced a more pronounced improvement in renal function and morphology, accompanied by reduced oxidative stress and a significant decrease in plasma KIM-1 levels, a sensitive marker of tubular injury.

These results suggest that combining the modulation of oxidative stress and AT2 receptor-mediated protective mechanisms may represent a promising therapeutic strategy for limiting renal damage during IRI. Moreover, this approach may have potential relevance in clinical settings associated with I/R AKI, including kidney transplantation. Although further experimental and clinical studies are required, the present findings provide a valuable basis for the development of future pharmacological interventions aimed at improving outcomes in vulnerable patients at risk of ischemic renal injury.

## Figures and Tables

**Table 1:** Systemic haemodynamic parameters, 24 h after reperfusion.

	SHAM *n* = 7	AKI *n* = 7	NOV *n* = 9	APO *n* = 9	COM *n* = 9
**MAP (mmHg)**	130.4 ± 5.8	96.1 ± 6.2 ***	122.2 ± 3.4 ^###^	111.6 ± 4.1 *^#^	109.9 ± 4.8 **^#^
**HR (beat/min)**	437.7 ± 7.4	390.1 ± 12.0 ***	377.4 ± 6.7 ***^&^	392.6 ± 9.8 **	402.4 ± 6.7 **
**CO (mlxkg/min)**	361.9 ± 48.9	326.4 ± 48.5	464.9 ± 48.0 ^#^	459.7 ± 65.6	533.0 ± 27.1 *^##^
**TPVR (mmHg × min/mL × kg)**	0.56 ± 0.10	0.48 ± 0.08	0.40 ± 0.04	0.37 ± 0.05 *	0.30 ± 0.10 **^#^

MAP—mean arterial pressure; HR—hearth rate; CO—cardiac output; TPVR—total peripheral vascular resistance; *n*—number of animals; SHAM—Sham-operated rats (model control group); AKI—control group with acute kidney injury; NOV—animals with AKI and received novokinin; APO—animals with AKI and received apocynin; COM—animals with AKI and received NOV and APO; * *p* < 0.05, ** *p* < 0.01, *** *p* < 0.001 vs. SHAM group; ^#^ *p* < 0.05, ^##^ *p* < 0.01, ^###^ *p* < 0.001 vs. AKI group; ^&^ *p* < 0.05 vs. COM group. The results are expressed as mean ± standard error of the mean.

**Table 2:** Correlation between KIM-1 levels and renal function parameters.

*n* = 41	Pcr	Pu	Pphos
**KIM-1**	r = 0.5931 ***p* = 0.000**	r = 0.7131 ***p* = 0.000**	r = 0.6264 ***p* = 0.000**

Significant positive correlation between KIM-1 and kidney function parameters. *n*—number of samples; Pcr—Plasma creatinine; Pu—Plasma urea; Pphos—Plasma phosphate; Correlations are significant at *p* < 0.05.

**Table 3:** Plasma TBARS levels and erythrocytes antioxidant enzymes activity in experimental groups.

	SHAM *n* = 7	AKI *n* = 7	NOV *n* = 9	APO *n* = 9	COM *n* = 9
**TBARS (nmol/mL)**	6.2 ± 0.46	11.2 ± 1.35 **	11.0 ± 1.23 **	11.3 ± 1.24 **	8.4 ± 0.64
**CAT (KU/g Hb)**	77.6 ± 3.9	60.5 ± 1.9 **	60.6 ± 2.7 **	54.8 ± 4.1 ***^&^	67.4 ± 1.6
**SOD (U/g Hb)**	109.1 ± 10.4	92.6 ± 15.8	120.9 ± 14.3	77.8 ± 9.1	103.3 ± 20.7
**GSH-Px (U/g Hb)**	37.1 ± 4.4	26.7 ± 4.4	21.5 ± 2.7 *	26.7 ± 5.4	21.8 ± 4.8 *
**GR (U/g Hb)**	5.8 ± 1.3	3.4 ± 0.9	4.8 ± 0.7	5.1 ± 0.7	4.2 ± 0.7

Thiobarbituric acid reactive substances level in plasma (TBARS); Erythrocytes catalase (CAT), superoxide dismutase (SOD), glutathione peroxidase (GSH-Px) and glutathione reductase (GR) activity—24 h after reperfusion. SHAM—Sham-operated rats (model control group); AKI—control group with acute kidney injury; NOV—animals with AKI and received novokinin; APO—animals with AKI and received apocynin; COM—animals with AKI and received NOV and APO; * *p* < 0.05, ** *p* < 0.01, *** *p* < 0.001 vs. SHAM group; ^&^ *p* < 0.05, vs. COM. The results are expressed as mean ± standard error of the mean.

## Data Availability

The original contributions presented in this study are included in the article. Further inquiries can be directed to the corresponding author.

## Abbreviations

The following abbreviations are used in this manuscript:

IRI	Ischemia–reperfusion injury
AKI	Acute kidney injury
AT2R	Angiotensin II type 2 receptor
SHAM	Sham-operated animals
SHR	Spontaneously hypertensive rats
KIM-1	Kidney injury molecule 1
I/R	Ischemia and reperfusion
ROS	Reactive oxygen species
Ang II	Angiotensin II
AT1R	Angiotensin II type 1 receptor
APO	Apocynin
NOV	Novokinin
COM	Combination
MAP	Mean arterial pressure
HR	Heart rate
CO	Cardiac output
TPVR	Total peripheral vascular resistance
RBF	Renal blood flow
RVR	Renal vascular resistance
CBF	Carotid blood flow
CVR	Carotid vascular resistance
Pcr	Plasma creatinine
Pu	Plasma urea
Pphos	Plasma phosphate
TBARS	Thiobarbituric acid reactive substances
CAT	Erythrocytes catalase
GR	Erythrocytes glutathione reductase
SOD	Erythrocytes superoxide dismutase
GSH-Px	Erythrocytes glutathione peroxidase
PAS	Periodic acid-Schiff
ANOVA	Analysis of variance
LSD	Least significant difference
2K1C	Two kidney one clip
